# PCW-1001, a Novel Pyrazole Derivative, Exerts Antitumor and Radio-Sensitizing Activities in Breast Cancer

**DOI:** 10.3389/fonc.2022.835833

**Published:** 2022-03-29

**Authors:** Minsung Kang, Navin Pandit, Ah-Young Kim, Suk Joon Cho, Young-Ju Kwon, Jiyeon Ahn, Kyu Myung Lee, Sangwook Wu, Jeong Su Oh, Kwan-Young Jung, Jae-Sung Kim

**Affiliations:** ^1^ Division of Radiation Biomedical Research, Korea Institute of Radiological and Medical Sciences, Seoul, South Korea; ^2^ Department of Medicinal Chemistry and Pharmacology, University of Science & Technology, Daejeon, South Korea; ^3^ Therapeutics & Biotechnology Division, Korea Research Institute of Chemical Technology, Daejeon, South Korea; ^4^ Radiological and Medico-Oncological Sciences, University of Science and Technology, Seoul, South Korea; ^5^ Research & Development (R&D) Center, Pharmcadd, Busan, South Korea; ^6^ Department of Integrative Biotechnology, Sungkyunkwan University, Suwon, South Korea

**Keywords:** pyrazole derivative, breast cancer, chemotherapy, radio-sensitizer, combination therapy

## Abstract

As pyrazole and its derivatives have a wide range of biological activities, including anticancer activity, the design of novel pyrazole derivatives has emerged as an important research field. This study describes a novel pyrazole derivative that exerts antitumor and radiosensitizing activities in breast cancer both *in vitro* and *in vivo*. We synthesized a novel pyrazole compound N,N-dimethyl-N’-(3-(1-(4-(trifluoromethyl)phenyl)-1H-pyrazol-4-yl)phenyl)azanesulfonamide (PCW-1001) and showed that it inhibited several oncogenic properties of breast cancer both *in vitro* and *in vivo*. PCW-1001 induced apoptosis in several breast cancer cell lines. Transcriptome analysis of PCW-1001-treated cells showed that it regulates genes involved in the DNA damage response, suggesting its potential use in radiotherapy. Indeed, PCW-1001 enhanced the radiation sensitivity of breast cancer cells by modulating the expression of DNA damage response genes. Therefore, our data describe a novel pyrazole compound, PCW-1001, with antitumor and radiosensitizer activities in breast cancer.

## Introduction

Breast cancer is the most common cancer in women worldwide (1, 2). After surgery, breast cancer is often treated with radiotherapy and chemotherapy. Radiotherapy is recommended for most patients for local control following both breast-conserving surgery and mastectomy (3–5). Because radiation treatment induces apoptosis of highly proliferative cancer cells by triggering DNA damage such as DNA double-strand breaks (6, 7), the susceptibility to radiation-induced DNA damage responses is closely associated with radiation sensitivity (8). Furthermore, resistance to radiotherapy, leading to the failure of local control, decreases the overall survival rate in patients with breast cancer (9, 10). Therefore, compounds that induce apoptosis of cancer cells by modulating DNA damage responses following irradiation are considered radiosensitizing anticancer agents. Several DNA-damaging anticancer agents induce not only apoptosis in cancer cells but also increase radiation sensitivity (11). Therefore, the development of radiosensitizers to enhance the radiation sensitivity of cancer cells can improve the efficacy of radiotherapy in breast cancer and the overall outcome after treatment.

Pyrazole is an aromatic heterocyclic compound characterized by a five-membered ring composed of three carbon atoms and two nitrogen atoms in adjacent positions, as represented by the molecular formula C_3_H_4_N_2_. Pyrazole and its derivatives have been synthesized for a wide range of biological activities, including antimicrobial, antifungal, anti-inflammatory, anti-cancer, neuroprotective, and anti-viral activity (12–14). Certain Food and Drug Administration (FDA)-approved drugs, such as celecoxib, deracoxib, etoricoxib, and atorivodine, possess pyrazole scaffolds as the efficacy functional moiety (15–17). With this background, we herein introduce a sulfonylurea functional group to increase charge–charge interactions and hydrogen bonding interactions between the compound and the amino acid residue of the target protein.

In the current study, we report PCW-1001, a novel pyrazole compound, N,N-dimethyl-N’-(3-(1-(4-(trifluoromethyl)phenyl)-1H-pyrazol-4-yl)phenyl)azanesulfonamide, which exhibits anticancer activity in breast cancer models *in vitro* and *in vivo*. Furthermore, this study showed that PCW-1001 induces apoptosis and the DNA damage response, thereby increasing the radiation sensitivity of breast cancer cells. Therefore, our data suggest that PCW-1001 is a novel pyrazole compound that exerts antitumor and radiosensitizer activity in breast cancer.

## Materials And Methods

### Cell Culture and Treatment

All cell lines were purchased from American Type Culture Collection (ATCC; Manassas, VA, USA). The cell lines were passaged for less than 2 months as ATCC protocols, and mycoplasma infection was checked by PCR once a week. Growth medium for BT549, MDA-MB-453, MCF7, and T47D cells were Dulbecco’s modified Eagle medium (DMEM; Corning, NY, USA), and MDA-MB-231 cells were maintained in RPMI 1640 (Welgene, Daegu, South Korea) supplemented with 10% fetal bovine serum (Corning) and 1% penicillin/streptomycin (GenDEPOT, USA) or 1% ZellShield™ (Minerva Biolabs, Berlin, Germany). The cells maintained under standard conditions, at humidified atmosphere of 5% CO2 at 37°C.

### Chemistry

PCW-1001 was synthesized by the following procedure at the Korea Research Institute of Chemical Technology (Daejeon, South Korea). Unless otherwise stated, all reactions were performed under an inert (N_2_) atmosphere. Reagents and solvents were reagent grade and purchased from Sigma-Aldrich, Alfa Aesar, and TCI Tokyo. Flash column chromatography was performed using silica gel 60 (230–400 mesh, Merck) with the indicated solvents. Thin-layer chromatography was performed using 0.25 mm silica gel plates. Proton nuclear magnetic resonance (^1^H NMR) spectra were recorded on a BRUKER ultra-shield 300 MHz NMR spectrometer at 25°C. Chemical shifts are reported in parts per million (ppm). ^1^H NMR data are reported as follows: chemical shift (δ ppm) (multiplicity, integration, coupling constant [Hz]). Multiplicities are reported as follows: s = singlet, d = doublet, t = triplet, q = quartet, and m = multiplet. The residual solvent peak was used as an internal reference. The mass spectra were obtained using Acuity™ waters A06UPD9BM and Agilent Technologies SG12109048. Prior to biological testing, the final compounds were confirmed to be > 95% pure by ultra-performance liquid chromatography (UPLC) using a Waters ACQUITY H-class system fitted with a C18 reversed-phase column (ACQUITY UPLC BEH C18: 2.1 mm × 50 mm, Part no. 186002350) according to the following conditions: (A) H_2_O + 0.1% formic acid, (B) CH_3_CN + 0.1% formic acid, (C) methanol (MeOH) + 0.1% formic acid; (I) a gradient of 95% A to 95% B over 5 min; (II) a gradient of 95% A to 95% C over 5 min.

### 4-bromo-1-(4-(trifluoromethyl)phenyl)-1H-pyrazole (3)

4-Iodobenzotrifluoride (5.00 g, 18.4 mmol) and 4-bromopyrazole (3.18 g, 22.1 mmol) were dissolved in anhydrous N,N-dimethylformamide (DMF; 50 mL). Cs_2_CO_3_ (13.4 g, 41.3 mmol) and Cu(OAc)_2_ (117 mg, 0.64 mmol) were added to the reaction flask. The reaction mixture was stirred at 110°C for 24 h, monitoring by TLC (eluent condition: 50% EtOAc in hexane). The reaction was quenched by adding H_2_O (100 mL) and partitioned with Et_2_O (400 mL). The diethylether (Et_2_O) layer was washed with H_2_O (3 × 50 mL) and brine (50 mL). The organic layer was collected, dried over anhydrous Na_2_SO_4_, filtered, concentrated, and purified by silica column chromatography (20% EtOAc in hexane) to obtain compound 3 in 78.5% (4.21 g) yield as a brown solid. ^1^H NMR (300 MHz, CDCl_3_) δ 7.93 (s, 1H), 7.67 (s, 1H), 7.49 (d, 2H, *J* = 7.5 Hz), 7.34 (d, 2H, *J* = 7.5 Hz); mass spectrometry (MS electrospray ionization [ESI]) *m/z* Calcd for C_10_H_6_BrF_3_N_2_ (M^+^): 290.0, found: 291.1 (M+H^+^).

### 3-(1-(4-(trifluoromethyl)phenyl)-1H-pyrazol-4-yl)aniline (4)

Compound **3** (4.00 g, 13.7 mmol), 3-aminophenylboronic acid (2.45 g, 17.8 mmol), and K_3_PO_4_ (7.29 g, 34.3 mmol) were added to a reaction flask containing 1,4-dioxane (36 mL) and H_2_O (9 mL). The reaction mixture was stirred at room temperature for 5 min by bubbling N_2_ (g), and then sSPhos (704 mg, 1.37 mmol) and Pd(OAc)_2_ (154 mg, 0.68 mmol) were added. Next, the reaction mixture was stirred at 80°C for 2 h, cooled to room temperature, diluted with EtOAc (200 mL), and washed with brine (3 × 20 mL). The organic layer was collected, dried over anhydrous Na_2_SO_4_, filtered, concentrated, and purified by silica column chromatography (35% EtOAc in hexane) to afford compound 4 in 64.8% (2.71 g) yield as a pale-yellow solid. ^1^H NMR (300 MHz, CDCl_3_) δ 8.21 (s, 1H), 8.09 (s, 1H), 7.80 (d, 2H, *J* = 8.7 Hz), 7.77 (d, 2H, *J* = 8.7 Hz), 7.62 (s, 1H), 7.50 (d, 1H, *J* = 7.4 Hz), 7.44 (t, 1H, *J* = 7.7 Hz), 7.31 (d, 1H, *J* = 7.7 Hz), 4.40 (br s, 2H); MS (ESI) *m/z* Calcd for C_16_H_12_F_3_N_3_ (M^+^): 303.1, found: 304.1(M+H^+^).

### N-(3-(1-(4-(trifluoromethyl)phenyl)-1H-pyrazol-4-yl)phenyl)ethanesulfonamide (5a)

Compound 4 (1.00 g, 3.30 mmol) was dissolved in anhydrous pyridine (3 mL) and cooled to 0°C using an ice-water bath. Ethanesulfonyl chloride (307 µL, 3.30 mmol) was added dropwise and stirred at room temperature for 8 h. The reaction was quenched by adding H_2_O (30 mL) and partitioned with EtOAc (100 mL). The EtOAc layer was washed with a saturated NH_4_Cl solution (3 × 40 mL) and brine (40 mL). The organic layer was collected, dried over anhydrous Na_2_SO_4_, filtered, concentrated, and purified by silica column chromatography (30% EtOAc in hexane) to afford compound 5a in 72.1% (940 mg) yield as a light-yellow solid. ^1^H NMR (300 MHz, CDCl_3_) δ 8.20 (s, 1H), 8.04 (s, 1H), 7.84 (d, 2H, *J* = 8.5 Hz), 7.77 (d, 2H, *J* = 8.5 Hz), 7.43 (m, 1H), 7.34 (m, 2H), 7.06 (m, 1H), 6.52 (s, 1H), 4.13 (q, 2H, *J* = 8.1 Hz), 1.47 (t, 3H, *J* = 8.1 Hz); ^13^C-NMR (CDCl_3_, 75 MHz) δ 143.0, 141.5, 138.2, 137.1, 130.0, 128.5, 127.9, 125.5, 125.4, 124.1, 123.7, 123.5, 119.4, 117.5, 114.2, 52.9, 2.3; MS (ESI) *m/z* Calcd for C_18_H_16_F_3_N_3_O_2_S (M^+^): 395.1, found: 395.9 (M+H^+^).

### N-(3-(1-(4-(trifluoromethyl)phenyl)-1H-pyrazol-4-yl)phenyl) N,N-dimethylsulfonamide (5b)

The title compound **5b** was prepared from compound 4 (1.50 g, 4.95 mmol) and *N,N*-dimethylsulfamoyl chloride (520 µL, 3.79 mmol) in a manner similar to that described for compound 5a in 64.0% (1.31 g) yield as a light-yellow solid. ^1^H NMR (300 MHz, CDCl_3_) δ 8.21 (s, 1H), 8.01 (s, 1H), 7.89 (d, 2H, *J* = 8.5 Hz), 7.76 (d, 2H, *J* = 8.5 Hz), 7.40 (m, 1H), 7.36 (m, 2H), 7.09 (m, 1H), 6.54 (s, 1H), 2.89 (s, 6H); ^13^C-NMR (CDCl_3_, 75 MHz) δ 143.0, 141.4, 138.2, 137.1, 130.0, 128.5, 127.9, 125.5, 125.4, 125.2, 124.1, 123.7, 123.5, 119.4, 117.5, 114.2, 36.3(2C); MS (ESI) *m/z* Calcd for C_18_H_17_F_3_N_4_O_2_S (M^+^): 410.1, found: 410.9 (M+H^+^).

### N-methyl-N-(3-(1-(4-(trifluoromethyl)phenyl)-1H-pyrazol-4-yl)phenyl)ethanesulfonamide (6a)

Compound **5a** (300 mg, 0.76 mmol) was dissolved in MeCN (7 mL) and K_2_CO_3_ (314 mg, 2.27 mmol) was added, and the reaction mixture was stirred at room temperature for 20 min. A solution of iodomethane (236 µL, 3.79 mmol) in MeCN (1 mL) was added dropwise and stirred at 80°C for 2 h. The solvent was removed by evaporation, and the residue was dissolved in EtOAc (70 mL). The organic layer was washed with H_2_O (20 mL) and brine (20 mL). The organic layer was collected, dried over anhydrous Na_2_SO_4_, filtered, concentrated, and purified by silica column chromatography (20% EtOAc in hexane) to obtain compound 6a in 45% (142 mg) yield as a yellow solid. ^1^H NMR (300 MHz, CDCl_3_) δ 8.23 (s, 1H), 8.02 (s, 1H), 7.89 (d, 2H, *J* = 8.7 Hz), 7.76 (d, 2H, *J* = 8.7 Hz), 7.62 (s, 1H), 7.49 (d, 1H, *J* = 7.4 Hz), 7.45 (t, 1H, *J* = 7.7 Hz), 7.30 (d, 1H, *J* = 7.7 Hz), 3.40 (s, 3H), 3.12 (q, 2H, *J* = 7.4 Hz), 1.42 (t, 3H, *J* = 7.7 Hz); ^13^C-NMR (CDCl_3_, 75 MHz) δ 143.1, 141.5, 138.9, 138.8, 137.2, 130.1, 128.5, 127.9, 125.6, 125.5, 125.4, 124.1, 123.7(2C), 115.9, 111.4, 50.4, 32.7, 2.6; MS (ESI) *m/z* Calcd for C_19_H_18_F_3_N_3_O_2_S (M^+^): 409.1, found: 410.2 (M+H^+^).

### N-(4-chlorobenzyl)-N-(3-(1-(4-(trifluoromethyl)phenyl)-1H-pyrazol-4-yl)phenyl)ethanesulfonamide (6b)

Compound 6b was prepared from compound 5a (300 mg, 0.76 mmol) and 4-chlorobenzylbromide (234 mg, 1.14 mmol) in a manner similar to that described for compound 6a in 54.5% (215 mg) yield as a light-yellow solid. ^1^H NMR (300 MHz, CDCl_3_) δ 8.12 (s, 1H), 7.94 (s, 1H), 7.87 (d, 2H, *J* = 8.5 Hz), 7.75 (d, 2H, *J* = 8.5 Hz), 7.46 (m, 1H), 7.38 (m, 2H), 7.24 (m, 3H), 7.17 (m, 1H), 4.88 (s, 2H), 3.15 (q, 2H, *J* = 7.3 Hz), 1.47 (t, 3H, *J* = 7.3 Hz); ^13^C-NMR (CDCl_3_, 75 MHz) δ 143.0, 141.5, 139.7, 138.8, 138.7, 137.2, 132.3, 130.0, 129.3 (2C), 128.6, 128.5, 128.3, 127.9, 125.5, 125.4, 13.7, 123.6, 115.9, 111.4, 50.7, 50.5, 2.6; MS (ESI) *m/z* Calcd for C_25_H_21_ClF_3_N_3_O_2_S (M^+^): 519.1, found: 520.0 (M+H^+^).

### N-methyl-N-(3-(1-(4-(trifluoromethyl)phenyl)-1H-pyrazol-4-yl)phenyl)N,N-Dimethylsulfonamide (6c)

Compound **6c** was prepared from compound 5b (300 mg, 0.73 mmol) and iodomethane (236 µL, 3.66 mmol) in a manner similar to that described for compound 6a in 30.3% (94 mg) yield as a light-yellow solid. ^1^H NMR (300 MHz, CDCl_3_) δ 8.23 (s, 1H), 8.02 (s, 1H), 7.89 (d, 2H, *J* = 8.6 Hz), 7.76 (d, 2H, *J* = 8.6 Hz), 7.62 (m, 1H), 7.48 (m, 1H), 7.44 (t, 1H, *J* = 7.6 Hz), 7.32 (m, 1H), 3.33 (s, 3H), 2.83 (s, 6H); ^13^C-NMR (CDCl_3_, 75 MHz) δ 143.1, 141.5, 138.7, 138.6, 137.2, 130.0, 128.5, 127.9, 125.5 (2C), 125.4, 124.1, 123.7, 123.6, 115.9, 36.6 (2C), 30.3; MS (ESI) *m/z* Calcd for C_19_H_19_F_3_N_4_O_2_S (M^+^): 424.1, found: 425.0 (M+H^+^).

### N-(4-chlorobenzyl)-N-(3-(1-(4-(trifluoromethyl)phenyl)-1H-pyrazol-4-yl)phenyl)propane-2-sulfonamide (6d)

Compound 6d was prepared from compound 5b (300 mg, 0.73 mmol) and 4-chlorobenzylbromide (225 mg, 1.09 mmol) in a manner similar to that described for compound 6a in 48.6% (190 mg) yield as a yellow solid. ^1^H NMR (300 MHz, CDCl_3_) δ 8.12 (s, 1H), 7.94 (s, 1H), 7.88 (d, 2H, *J* = 8.8 Hz), 7.75 (d, 2H, *J* = 8.8 Hz), 7.46 (m, 1H), 7.37 (t, 1H, *J* = 7.6 Hz), 7.26 (s, 1H), 7.24 (d, 2H, *J* = 7.6 Hz), 7.15 (m, 1H), 4.78 (s, 2H), 2.78 (s, 6H); ^13^C-NMR (CDCl_3_, 75 MHz) δ 143.0, 141.5, 139.7, 138.8, 138.7, 137.2, 132.3, 130.0, 129.3, 129.2, 128.6, 128.5, 128.4, 128.2, 127.9, 125.5, 125.4, 125.2, 124.1, 123.7 (2C), 115.9, 114.1, 48.2, 36.6 (2C); MS (ESI) *m/z* Calcd for C_25_H_22_ClF_3_N_4_O_2_S (M^+^): 534.1, found: 535.1 (M+H^+^).

### 3-(1-(4-(trifluoromethyl)phenyl)-1H-pyrazol-4-yl)benzaldehyde (7)

Compound 3 (5.00 g, 17.2 mmol), 3-formylphenylboronic acid (3.35 g, 22.3 mmol) and Na_2_CO_3_ (4.55 g, 42.9 mmol) were added to the reaction flask containing 1,4-dioxane (40 mL) and H_2_O (10 mL). The reaction mixture was stirred at room temperature for 5 min by bubbling N_2_(g), and Pd(PPh_3_)_4_ (992 mg, 0.86 mmol) was added. Next, the reaction mixture was stirred at 80°C for 2 h, cooled to room temperature, diluted with EtOAc (200 mL), and washed with brine (3 × 20 mL). The organic layer was collected, dried over anhydrous Na_2_SO_4_, filtered, concentrated, and purified by silica column chromatography (30% EtOAc in hexane) to obtain compound 7 in 69.9% (3.80 g) yield as a light-brown solid. ^1^H NMR (300 MHz, CDCl_3_) δ 9.89 (s, 1H), 8.21 (s, 1H), 8.10 (s, 1H), 7.79 (d, 2H, *J* = 8.4 Hz), 7.77 (d, 2H, *J* = 8.4 Hz), 7.60 (s, 1H), 7.52 (d, 1H, *J* = 7.4 Hz), 7.41 (t, 1H, *J* = 7.4 Hz), 7.30 (d, 1H, *J* = 7.7 Hz); MS (ESI) *m/z* Calcd for C_17_H_11_F_3_N_2_O (M^+^): 316.1, found: 317.2 (M+H^+^).

### N-ethyl-1-(3-(1-(4-(trifluoromethyl)phenyl)-1H-pyrazol-4-yl)phenyl)methanimine oxide (8a)

Compound 7 (100 mg, 0.32 mmol), *N*-ethylhydroxylamine hydrochloride (92.5 mg, 0.96 mmol) and KOAc (93.1 mg, 0.96 mmol) were added to the reaction seal tube containing EtOH (3 mL). The reaction mixture was stirred at room temperature for 12 h. The reaction solvent was removed by evaporation, and the residue was dissolved in EtOAc (30 mL). The organic layer was washed with H_2_O (10 mL) and brine (10 mL). The organic layer was collected, dried over anhydrous Na_2_SO_4_, filtered, concentrated, and purified by prep-TLC (10% MeOH in dichloromethane) to obtain compound 8a in 83.6% (95 mg) yield as a light-yellow solid. ^1^H NMR (300 MHz, CDCl_3_) δ 9.04 (s, 1H), 8.72 (s, 1H), 7.91 (s, 1H), 7.54-7.40 (m, 7H), 4.07 (q, 2H, *J* = 7.1 Hz), 1.41 (t, 3H, *J* = 7.1 Hz); ^13^C-NMR (CDCl_3_, 75 MHz) δ 143.0, 141.5, 136.4, 130.9, 129.9, 129.1, 128.7, 128.5, 127.9, 127.5, 126.2, 125.5, 125.4, 125.3, 124.1, 123.7 (2C), 42.1, 16.0; MS (ESI) *m/z* Calcd for C_19_H_16_F_3_N_3_O (M^+^): 359.1, found: 360.2 (M+H^+^).

### N-isopropyl-1-(3-(1-(4-(trifluoromethyl)phenyl)-1H-pyrazol-4-yl)phenyl)methanimine oxide (8b)

Compound 8b was prepared from compound 7 (100 mg, 0.32 mmol) and *N*-isopropylhydroxylamine hydrochloride (105 mg, 0.96 mmol) in a manner similar to that described for compound 8a in 92.3% (109 mg) yield as a light-yellow solid. ^1^H NMR (300 MHz, CDCl_3_): δ 9.02 (s, 1H), 8.71 (s, 1H), 7.96 (s, 1H), 7.51-7.40 (m, 7H), 1.82 (m, 1H), 0.98 (d, 6H, J= 6.8 Hz); ^13^C-NMR (CDCl_3_, 75 MHz) δ 143.1, 141.5, 136.4, 130.8, 129.8, 129.1, 128.7, 128.5, 127.9, 127.4, 126.2, 125.5, 125.4, 125.1, 124.1, 123.7 (2C), 65.3, 20.5 (2C); MS (ESI) *m/z* Calcd for C_20_H_18_F_3_N_3_O (M^+^): 373.1, found: 374.0 (M+H^+^).

### N-tert-butyl-1-(3-(1-(4-(trifluoromethyl)phenyl)-1H-pyrazol-4-yl)phenyl)methanimine oxide (8c)

The title compound **8c** was prepared from compound 7 (100 mg, 0.32 mmol) and N-tert-butylhydroxylamine hydrochloride (119 mg, 0.96 mmol) in a manner similar to that described for compound 8a in 89.8% (110 mg) yield as a light-yellow solid. ^1^H NMR (300 MHz, CDCl_3_): δ 9.08 (s, 1H), 8.75 (s, 1H), 7.91 (s, 1H), 7.51-7.44 (m, 7H), 1.08 (s, 9H); ^13^C-NMR (CDCl_3_, 75 MHz) δ 143.0, 141.5, 136.4, 130.8, 129.8, 129.0, 128.7, 128.5, 127.8, 127.5, 126.1, 125.5 (2C), 125.4, 124.1, 123.7, 123.5, 70.7, 28.3 (3C); MS (ESI) *m/z* Calcd for C_21_H_20_F_3_N_3_O (M^+^): 387.2, found: 388.1 (M+H^+^).

### 3-(1-(4-(trifluoromethyl)phenyl)-1H-pyrazol-4-yl)benzaldehyde O-ethyl oxime (9a)

Compound 7 (100 mg, 0.32 mmol), *O*-ethylhydroxylamine hydrochloride (46.3 mg, 0.47 mmol) and pyridine (56 µL, 0.70 mmol) were added to the reaction seal tube containing DCM (3 mL). The reaction mixture was stirred at room temperature for 12 h. The reaction solvent was removed by evaporation, and the residue was dissolved in EtOAc (30 mL). The organic layer was washed with 1 N aqueous HCl (10 mL) and brine (10 mL). The organic layer was collected, dried over anhydrous Na_2_SO_4_, filtered, concentrated, and purified by prep-TLC (10% MeOH in DCM) to obtain compound 9a in 85.7% (105 mg) yield as a pale orange solid. ^1^H NMR (300 MHz, CDCl_3_): δ 9.08 (s, 1H), 8.61 (s, 1H), 7.65 (s, 1H), 7.47-7.41 (m, 7H), 4.10 (q, *J* = 6.8 Hz, 2H), 1.36 (t, *J* = 6.8 Hz, 3H); ^13^C-NMR (CDCl_3_, 75 MHz) δ 153.8, 143.0, 141.5, 136.5, 134.2, 129.8, 129.6, 129.2, 128.5, 127.9, 126.3, 125.5, 125.4, 124.1, 123.7 (2C), 125.5, 68.2, 12.8; MS (ESI) *m/z* Calcd for C_19_H_16_F_3_N_3_O (M^+^): 359.1, found: 360.0 (M+H^+^).

### 3-(1-(4-(trifluoromethyl)phenyl)-1H-pyrazol-4-yl)benzaldehyde O-(tert-butyl) oxime (9b)

The title compound **9b** was prepared from compound **7** (100 mg, 0.32 mmol) and *O-tert*-butylhydroxylamine hydrochloride (59.6 mg, 0.47 mmol) in a manner similar to that described for compound 9a in 89.0% (109 mg) yield as a light-orange solid. ^1^H NMR (300 MHz, CDCl_3_): δ 9.06 (s, 1H), 8.66 (s, 1H), 7.64 (s, 1H), 7.46-7.40 (m, 7H), 1.16 (s, 9H); ^13^C-NMR (CDCl_3_, 75 MHz) δ 153.7, 143.0, 141.4, 136.5, 134.1, 129.7, 129.5, 129.1, 128.5, 127.8, 126.3, 125.5, 125.3, 124.0, 123.7, 123.6, 125.5, 93.5, 31.2 (3C); MS (ESI) *m/z* Calcd for C_21_H_20_F_3_N_3_O (M^+^): 387.2, found: 388.1 (M+H^+^).

### Cell Viability Assay

Cell viability was determined indirectly using the WST-8 assay (Cyto X™ cell viability assay kit; LPS solution, Daejeon, South Korea), which measures metabolic capacity, in accordance with the manufacturer’s protocol. Briefly, cells were plated in a 96-well plate and treated with PCW-1001. After 72 h, the old medium was removed, a colorimetric reagent was added, and absorbance was measured.

### Clonogenic Assay

Cell survival after irradiation was determined using a clonogenic assay (18). Five hundred to one thousand cells were plated in a 60 mm plate and incubated with or without PCW-1001 for 10 days. The colonies were stained with a crystal violet solution, and the number of colonies was counted using ImageJ, downloaded from imagej.nih.gov/ij. Survival faction was calculated based on previous research (19).

### Sphere Formation Assay

A sphere formation assay was performed as previously reported (20, 21). Briefly, cells were plated in an ultra-low attachment plate (Corning, NY) and cultured in serum-free DMEM-F12 medium (Gibco Laboratories, Grand Island, NY) supplemented with 1:50 B-27 (Invitrogen, Carlsbad, CA), 20 ng/mL FGF (R&D Systems, Minneapolis, MN), and 20 ng/mL EGF (R&D Systems) and treated with or without PCW-1001. After 10 days, the diameter and number of spherical colonies were analyzed using ImageJ software.

### Xenograft Studies

Five-week-old female BALB/c nude mice were purchased from Orient Bio (Seongnam, Korea) and maintained under aseptic conditions for a week. Mice were maintained under a 12-hour day-night cycle and *ad libitum* feeding. BT549 cells (5 × 10^6^ cells) were implanted into the inguinal mammary fat pad. Five days after injection, the mice were randomized into two groups: Ctrl and PCW-1001. PCW-1001 was formulated in 10% dimethylsulfoxide (DMSO), 1% Tween-20, and 89% saline, and 30 mg/kg of PCW-1001 or 10% DMSO was administered by subcutaneous injection twice a week. Tumor diameter and body weight were measured three times per week. The Korea Institute of Radiological and Medical Sciences (KIRAMS) Institutional Animal Care and Use Committee (IACUC) approved all animal processes and treatment (Kirams2018-0062).

### Annexin V Assay

Cell apoptosis was determined using the FITC Annexin V Apoptosis Detection Kit II (BD Biosciences, San Jose, CA, USA) in accordance with the manufacturer’s protocol.

### Western Blot Analysis

Western blotting was performed as previously described (18, 22). Briefly, the proteins were separated by sodium dodecyl sulfate (SDS-) polyacrylamide gel electrophoresis, transferred to a polyvinylidene fluoride (PVDF) membrane, and detected using specific antibodies. The following antibodies were used: rabbit monoclonal antibodies against chk2 (6334S; Cell Signaling Technology), Ki-67 (GTX16667; GeneTex, CA, USA), survivin (2808S; Cell Signaling Technology), and pro-caspase 3 (#9664; Cell Signaling Technology), mouse monoclonal antibodies against β-actin (sc-47778; Santa Cruz Biotechnology) and pro-caspase 9 (551246; BD Pharmingen), rabbit polyclonal antibodies against cleaved PARP (Asp214) (#9541; Cell Signaling Technology), and phospho-Chk2 (Thr68) (#2661; Cell Signaling Technology). The blots were developed using peroxide-conjugated secondary antibodies and enhanced by a chemiluminescence detection system (GE Healthcare Life Sciences, Little Chalfont, UK). Images of the bands were obtained using an Amersham Imager 600 system (GE Healthcare Life Sciences), and the intensity of the bands was quantified using ImageJ software.

### Immunofluorescence

Immunofluorescence analysis was performed as described previously (22, 23). Briefly, the cells were fixed with 4% paraformaldehyde, permeabilized with 0.1% Triton X-100, and blocked with 1% fetal calf serum in phosphate-buffered saline (PBS). The fixed cells were consecutively incubated with primary antibodies against γ-H2AX (1:100; EMD Millipore Corp., CA, USA) and secondary antibodies (anti-mouse Alexa-594; 1:400; Invitrogen). The cells were treated with Hoechst 33342 in 1× PBS, and images of γ-H2AX were obtained using an IN Cell Analyzer 6000 (GE Healthcare Life Sciences).

### Quantitative Real-Time PCR

Quantitative real-time-polymerase chain reaction (qRT-PCR) was performed as previously described (22). The following primer sequences were used: *β-actin* forward, 5’-CATGTACGTTGCTATCCAGGC-3; *β-actin* reverse, 5’-CTCCTTAATGTCACGCACGAT-3’; *ddit-3* forward, 5’-GCGCATGAA GGAGAAAGAAC-3’; *ddit-3* reverse, 5’-TCACCATTCGGTCAATCAGA-3’; *ddit4* forward 5’-CGAGTCCCTGGACAGCAG-3’ *ddit-4* reverse, 5’-GGTCACTGAGCAGCTCGAAG-3’; *bdnf* forward 5’-TAACGGCGGCAGACAAAAAGA-3’; *bdnf* reverse 5’-GAAGTATTGCTTCAGTTGGCCT-3’; *fen-1* forward 5’-AAGGTCACTAAGCAGCACAATG-3; *and fen-1* reverse 5’-GTAGCCGCAGCATAGACTTG-3’. A LightCycler ^®^ 96 (Roche Life Science) was used for RT-PCR. The gene expression was determined using the 2^–ΔΔ^Ct method, and *β-actin* was used as an internal control for normalization.

### Gene Expression Analysis

Gene expression analysis of PCW-1001 was performed using the nCounter multiplex gene expression analysis system (NanoString Technologies, WA, USA). MCF7 cells were treated with DMSO and 10 μM PCW-1001 for 24 h, and afterward, cell lysates were analyzed using the nCounter^®^PanCancer Pathways Panel (770 genes) in accordance with the manufacturer’s protocol. Quantitative changes in mRNA levels were analyzed and clustered using the nSolver software (NanoString Technologies).

### Statistical Analysis

A two-tailed Student’s *t*-test was performed to analyze the statistical differences between the groups. Statistical significance was set at *P* < 0.05. Statistical analyses were performed using Excel and XLSTAT software. To confirm that we had enough samples for statistical testing, G-Power 3.1 was used to perform a *post-hoc* power analysis (https://download.cnet.com/s/g-power).

## Results

### Synthesis of PCW-1001

**Figure 1:**
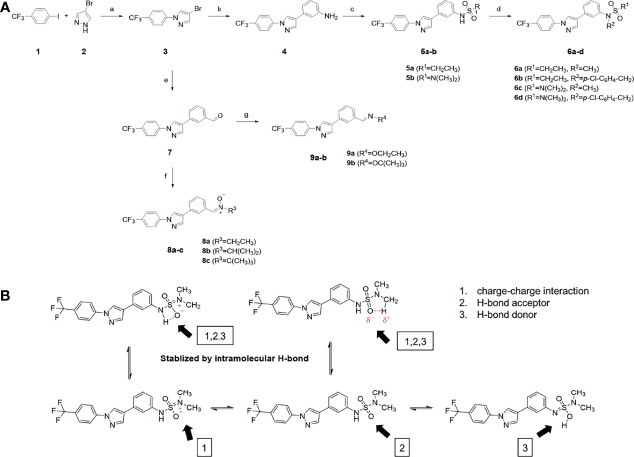
Synthesis of PCW-1001. **(A)** Reaction scheme to synthesize 11 different compounds containing pyrazole group. aReagents and conditions: (a) Cu(OAc)2, Cs2CO3, DMF, 110°C, 24 h. (b) Pd(OAc)2, K3PO4, sSPhos, dioxane, H2O, 80°C, 2 h. (c) Pyridine, 0°C to rt. 8 h (d) K2CO3, 80°C, 2 h. (e) Pd(PPh3)4, Na2CO3, dioxane, H2O, 80°C, 2 h. (f) KOAc, EtOH, room temperature, 2 h. (g) Pyridine, room temperature, 2 h. **(B)** The N,N-dimethylsulfonyl urea group exhibit charge–charge interaction, hydrogen bonding acceptor, and hydrogen bonding donor characteristics.

The synthetic routes and chemical structures of the 1-(4-(trifluoromethyl)phenyl)-1H-pyrazole compounds (5a–5b, 6a–6d, 8a–8b, 9a–9b) are shown in **Figure 1A**. Starting materials 1 and 2 were obtained from commercial suppliers and used after confirming their purity by liquid chromatography (LC)-mass analysis. The C─N coupling between compounds 1 and 2 was conducted in the presence of copper(II) acetate to obtain compound 3. Boronic acids and organohalides act as coupling partners in the Suzuki coupling reaction for C–C bond formation (24). Using this well-known reaction, compounds 3 and (3-aminophenyl)boronic acid were reacted in the presence of a palladium (0) complex, yielding intermediate 4. Analogous sulfonamide 5 was prepared using a similar synthetic strategy following the amide bond formation of intermediate 4 in pyridine with ethanesulfonyl chloride or *N,N*-dimethylsulfonyl chloride. The R^1^ functional group was introduced to the nitrogen atom of the SO_2_NH group to afford compounds 6a–6b. *N*-oxide analogs were obtained from the initial coupling of (3-formylphenyl)boronic acid with compound 3 to obtain intermediate 7, which was subsequently reacted with *N*-substituted hydroxylamine hydrochloride in the presence of potassium acetate as an inorganic base to yield 8a–8c. Oxime-substituted pyrazole compounds 9a–9b were synthesized from intermediate 7 and *O*-substituted hydroxylamine hydrochloride. To assess antitumor activity, cell viability assays were performed on multiple cancer cell lines, including breast, brain, colon, and lung cancer cells, with 20 µM of 11 synthesized novel compounds. Among the 11 compounds, compound 5b showed the most effective antitumor activity in multiple cancer cell lines (**Table 1**). The sulfonylurea functional group of compound 5b was beneficial for interaction with the target protein (**Figure 1B**). Due to the presence of a dipole moment among the sulfonylurea moiety, it exhibits several kinds of interactions, such as charge–charge interaction, H-bond acceptor, and donor characteristics (**Figure 1B**). In particular, the H-bond acceptor and donor characteristics of sulfonylurea exist as inter-and intramolecular hydrogen bonds with the available adjacent hydrogen atom (25, 26). In this study, compound 5b, *N,N*-dimethyl-*N’*-(3-(1-(4-(trifluoromethyl)phenyl)-1H-pyrazol-4-yl)phenyl)azanesulfonamide, was named PCW-1001.

**Table 1:** Cell viability analysis of pyrazole derivatives in multiple cancer cell lines.

No.	Structure	Molecular weight	MCF7(20 μM)	U373(20 μM)	LN18(20 μM)	DLD-1(20 μM)	HCT116(20 μM)	A549(20 μM)	H1299(20 μM)
**5a**	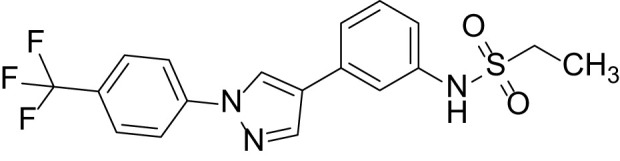	395.40	33.01	14.17 ± 2.92	7.52 ± 0.22	10.9 ± 2.67	5.42 ± 4.86	20.62 ± 12.69	6.93 ± 2.63
**5b** (PCW-1001)	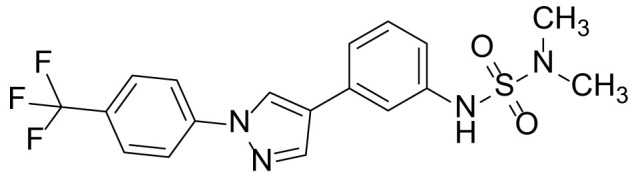	410.42	10.07 ± 10.01	8.71 ± 2.09	5.36 ± 0.49	7.93 ± 2.52	1.99 ± 0.81	14.69 ± 0.58	8.75 ± 6.86
**6a**	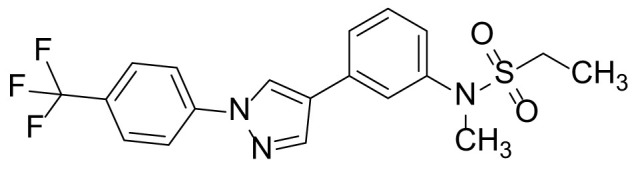	409.43	63.12 ± 4.04	48.21 ± 7.59	8.96 ± 1.16	12.48 ± 2.31	42.02 ± 2.85	68.63 ± 2.07	77.64 ± 8.26
**6b**	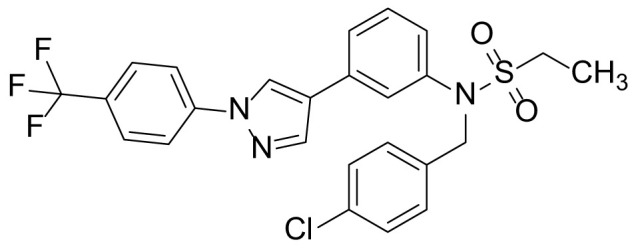	519.98	75.19 ± 15.33	104.45 ± 20.67	133.12 ± 13.04	109.24 ± 5.48	91.02 ± 6.71	153.97 ± 1.93	173.18 ± 17.17
**6c**	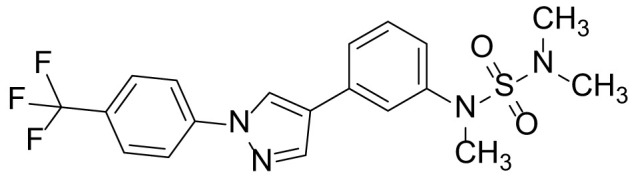	424.45	66.67 ± 3.7	46.02 ± 5.6	8.81 ± 1.15	9.77 ± 0.47	59.94 ± 3.31	90.42 ± 12.87	75.54 ± 7.2
**6d**	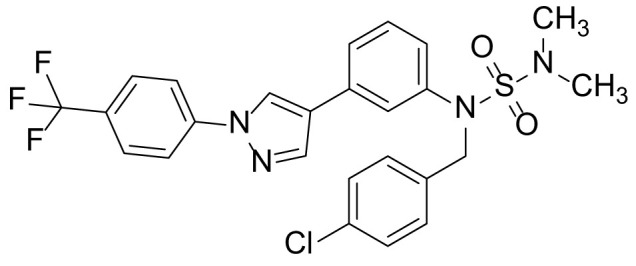	534.99	91.92 ± 1.48	67.44 ± 10.33	97.12 ± 0.23	82.68 ± 1.66	46.74 ± 6.94	109.21 ± 23.2	113.04 ± 6.93
**8a**	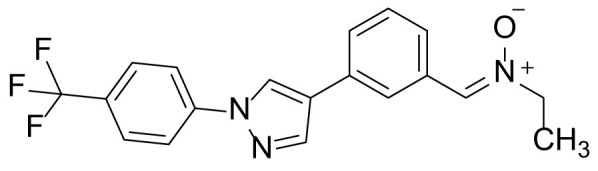	359.35	71.30 ± 29.98	72.10 ± 20.27	74.33 ± 7.37	62.66 ± 13.37	46.07 ± 8.68	89.69 ± 22.88	101.81 ± 3.66
**8b**	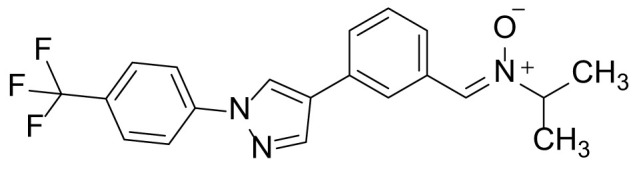	373.38	67.79 ± 35.61	65.09 ± 12.03	64.39 ± 6.64	40.25 ± 11.49	42.74 ± 1.39	86.63 ± 3.07	102.16 ± 6.03
**8c**	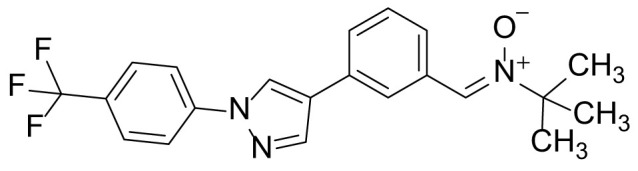	387.41	75.31 ± 29.06	68.12 ± 17.42	54.23 ± 9.92	34.83 ± 4.01	35.81 ± 0.99	93.43 ± 14.71	95.35 ± 1.78
**9a**	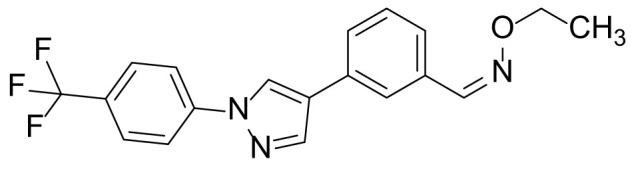	359.35	93.22 ± 14.36	96.66 ± 8.6	78.99 ± 11.63	76.95 ± 9.51	58.38 ± 6.74	135.48 ± 27.73	96.14 ± 1.57
**9b**	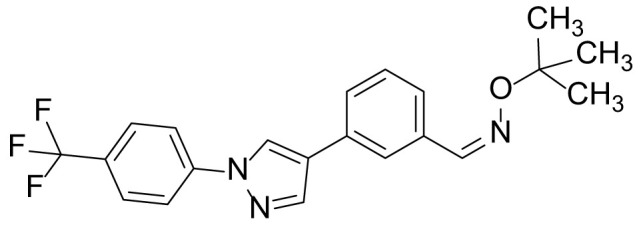	387.41	59.31 ± 2.8	92.20 ± 7.8	54.70 ± 6	54.59 ± 13.65	39.28 ± 8.65	70.38 ± 19.33	75.7 5 ± 1.57

### PCW-1001 Exerts Anticancer Activities in Breast Cancer *In Vitro* and *In Vivo*

**Figure 2:**
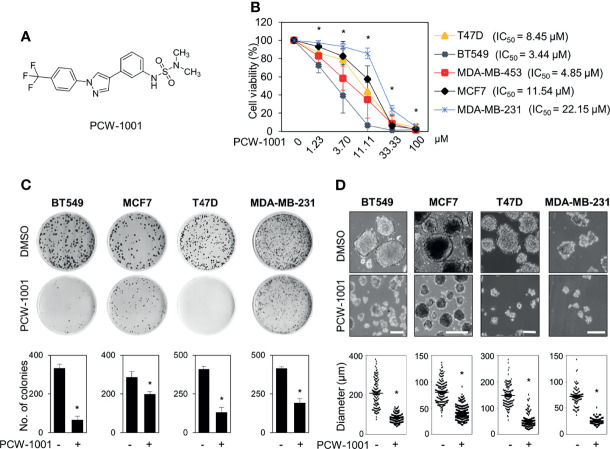
PCW-1001 inhibited several oncogenic properties of breast cancer cells. **(A)** The chemical structure of PCW-1001. **(B)** Breast cancer cell lines, including T47D, BT549, MDA-MB-231, MCF7, and MDA-MB-453, were treated with the indicated concentration of PCW-1001 for 72 h, and cell viability was analyzed. **(C, D)** The same cell lines were treated with 10 μM PCW-1001 for 14 days, and colony formation **(C)** and mammosphere formation **(D)** were analyzed. Upper panels are representative images and lower graphs are quantification data using ImageJ software or DIXI image solution. Scale bars = 100 µm. The data represent the results and are presented as mean ± standard deviation of three independent experiments. *P < 0.01.

**Figure 3:**
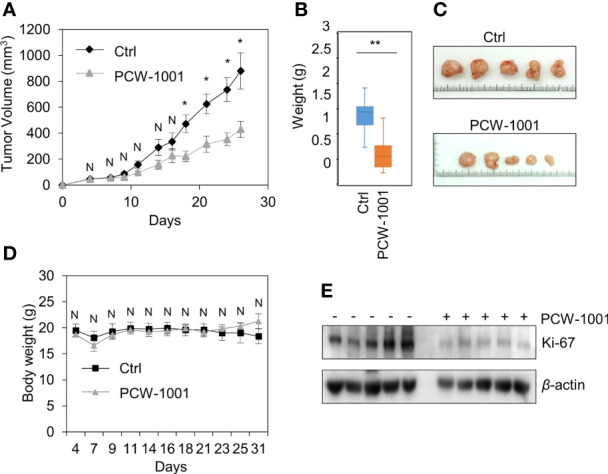
PCW-1001 reduced tumor growth of breast cancer cells *in vivo*. Female BALB/c nude mice were subcutaneously injected with 5 × 10^6^ BT549 cells and treated with DMSO (Ctrl) or PCW-1001 (30 mg/kg) twice a week. **(A–D)** The tumor volume **(A)** and body weight **(D)** were measured periodically as indicated, and the tumor weights **(B)** and images **(C)** were obtained at the end of the experiment. **(E)** Cell lysates of the tumor were analyzed by immunoblotting with an anti-Ki67 antibody. β-actin was used as the loading control. The data represent the results and are presented as mean ± standard deviation of three independent experiments. N, not significant; *P < 0.05, **P < 0.01.

Because breast cancer is the most common cancer in women, we next examined the antitumor activity of PCW-1001 in breast cancer cell lines using several approaches, including cell viability, clonogenic, and mammosphere formation assays. Cell viability analysis showed that PCW-1001 inhibited the viability of several breast cancer cell lines in a dose-dependent manner (**Figures 2A, B**). The half-maximal inhibitory concentration (IC_50_) values of T47D, BT549, MDA-MB-453, MCF7, and MDA-MB-231 cell lines were 8.45, 3.44, 4.85, 11.54, and 22.15 μM, respectively. In addition, we found that PCW-1001 (10 μM) reduced colony formation and mammosphere formation in T47D, BT549, MCF7, and MDA-MB-231 cells (**Figures 2C, D**), indicating that PCW-1001 has *in vitro* anticancer activity in breast cancer cells. Next, the *in vivo* antitumor effect of PCW-1001 was examined using the BT549 xenograft mouse model. Consistent with the *in vitro* data, PCW-1001 treatment (30 mg/kg) reduced tumor growth in the BT549 xenograft model (**Figures 3A** **–C**) without notable changes in body weight (**Figure 3D**), suggesting the absence of gross toxicity in the treated mice. In addition, reduced protein expression of Ki-67, a marker of cell proliferation, confirmed the inhibition of BT549 cell growth (27) (**Figure 3E**). Therefore, our results indicate that PCW-1001 has antitumor activity in breast cancer both *in vitro* and *in vivo.*

### PCW-1001 Induces Apoptosis of Breast Cancer Cells

**Figure 4:**
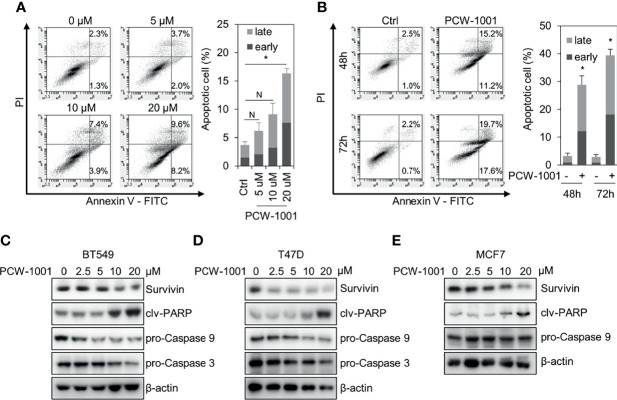
PCW-1001 induced apoptosis of breast cancer cell lines. Annexin-V assay was performed with BT549 cells treated with the indicated doses of PCW-1001 for 24 h **(A)** or 10 μM PCW-1001 for 48 or 72 h **(B)** to study apoptosis. **(C–E)** BT549, T47D, and MCF7 cells were treated with increasing doses of PCW-1001 for 24 h. Cell lysates were analyzed by immunoblotting with the indicated antibodies. β-actin was used as the loading control. The data represent the results and are presented as mean ± standard deviation of three independent experiments. N, not significant; *P < 0.01.

As the induction of apoptosis is a general mechanism of anticancer drugs (28, 29), we examined whether PCW-1001 induces apoptosis in breast cancer cells. Annexin V analysis showed that PCW-1001 induced apoptosis of BT549 cells in a dose- and time-dependent manner (**Figures 4A, B**). PCW-1001 also induced G1 cell cycle arrest, indicating that it disrupted the cell cycle (**Supplementary Figure 1A**). In addition, we observed that PCW-1001 decreased the expression of survivin, a member of the inhibitor of apoptosis protein (IAP) family, that inhibits caspases and blocks cell death (30, 31), whereas PCW-1001 increased the expression of cleaved-PARP and decreased that of pro-caspase 9 and pro-caspase 3, apoptosis markers (32, 33), in BT549, T47D, and MCF7 cells (**Figures 4C–E**). However, we could not confirm the change in pro-caspase 9 expression in MCF7 cells, which do not express caspase 3 (34). Altogether, these results demonstrated that PCW-1001 induced apoptosis in breast cancer cells.

### PCW-1001 Radio-Sensitizes Breast Cancer Cells by Modulating DNA Damage Response

**Figure 5:**
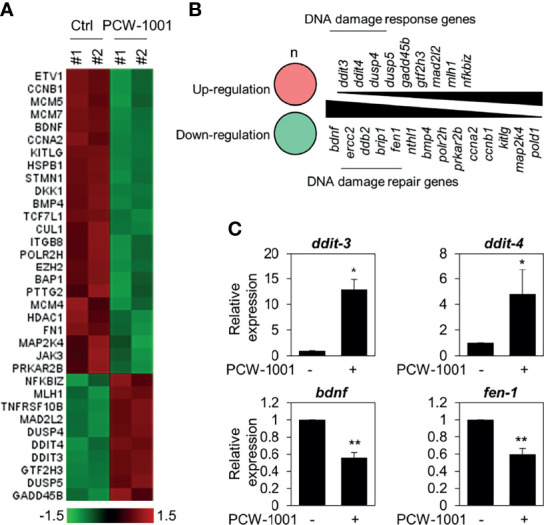
Gene expression analysis in PCW-1001-treated MCF7 cells. MCF7 was treated with DMSO (Ctrl) and 10 μM PCW-1001 for 24 h. The mRNA expression was analyzed using the nCounter^®^PanCancer Pathway Panel that was designed to quantitate 770 genes. A heatmap of upregulated (red) and downregulated (green) genes **(A)** and lists of upregulated DNA damage response genes **(B)**, upper panel) and down-regulated DNA damage repair genes **(B)**, lower panel) were analyzed based on the database. Genes exhibited changes more than 1.5-fold. **(C)** Cell lysates were analyzed by qPCR. GAPDH was used as an internal control. The data represent the results and are presented as mean ± standard deviation of three independent experiments. *P < 0.05, **P < 0.01.

To understand the molecular mechanism of PCW-1001-induced apoptosis, we performed gene expression analysis using the nCounter^®^PanCancer Pathways Panel in MCF7 breast cancer cells. MCF7 is one of the most common cell lines used to study breast cancer (35); further, PCW-1001 showed clear anti-cancer effects in MCF7, similar to those in the other cell lines, BT549 and T47D, used in this study. Therefore, we used MCF7 as a representative for the panel study. Interestingly, we found that PCW-1001 treatment upregulated DNA damage response genes and downregulated DNA damage repair genes (**Figures 5A, B**). We confirmed the upregulation of DNA damage response genes, such as DNA damage-inducible transcript (*DDIT*)-*3* (also known as *CHOP*) and -4 (also known as *REDD1*) (36, 37), and downregulation of DNA damage repair genes, such as brain-derived neurotrophic factor (*BDNF*) and flap structure-specific endonuclease 1 (*FEN1*) (38, 39) (**Figure 5C**), by qRT-PCR. These results suggested that PCW-1001 modulated the DNA damage response in breast cancer cells.

**Figure 6:**
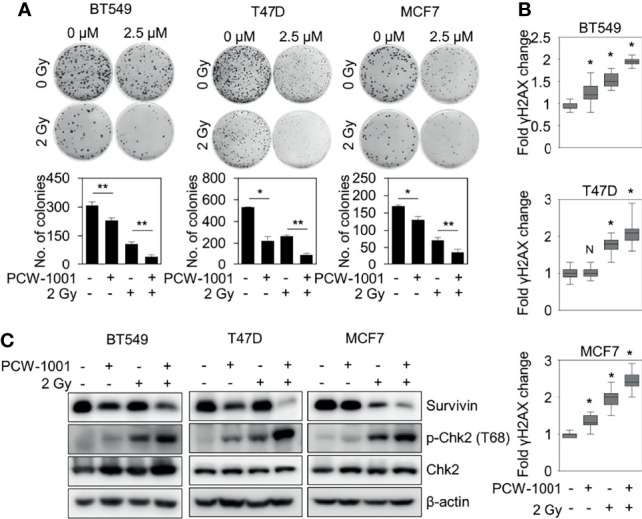
PCW-1001 enhanced the radiation sensitivity of breast cancer cells. BT549, T47D, and MCF7 cells were treated with DMSO (Ctrl) and PCW-1001 (**A**; 2.5 μM, **B, C**; 10 μM) without or with 2 Gy radiation for 14 days, 1 h, or 24 h. **(A)** Colony formation was determined using the colony formation assay; representative images (upper panel) and the quantification (lower panel) of colony formation of each cell line treated with indicated conditions. **(B)** Immunofluorescent staining was performed to measure the level of γH2AX, and the intensities of γH2AX were quantified using the IN Cell Analyzer HCA System. **(C)** Cell lysates were analyzed by immunoblotting with the indicated antibodies. β-actin was used as the loading control. The data represent the results and are presented as mean ± standard deviation of three independent experiments. N, not significant; *P < 0.05; **P < 0.01.

Because our data suggested that PCW-1001 regulated DNA damage response, we next investigated whether PCW-1001 could improve radiation sensitivity in breast cancer cells. Our clonogenic analysis revealed that PCW-1001 enhanced the radiation sensitivity of several breast cancer cells in response to 2 Gy irradiation (**Figure 6A**). Survival faction, calculated based on the data, showed the stronger effect of the combination of PCW-1001 and irradiation than a single treatment (**Supplementary Table 1**). The combination index of PCW-1001 and irradiation in breast cancer cells also ranged from moderate and slight to very strong synergism (**Supplementary Figure 2A**). In addition, the combination of PCW-1001 and irradiation increased the number of γ-H2AX foci, a biomarker for evaluating the efficacy of radiation-modifying compounds (40) (**Figure 6B** and **Supplementary Figure 2B**). Furthermore, the combination of PCW-1001 and radiation increased the levels of phospho-chk2, a DNA damage checkpoint protein (41) (**Figure 6C**). Chk-2 arrests the cell cycle at several checkpoints including G1/S when DNA is damaged, and activates p53, which induces p53-dependent apoptosis (42). The combination treatment also decreased the expression of survivin, an important biomarker of apoptosis and mitosis and a therapeutic target in various cancers (43) (**Figure 6C**), which is used in the diagnosis and prognosis of breast cancer (44). Therefore, our results collectively suggested that PCW-1001 enhanced the radiation sensitivity of breast cancer cells by modulating DNA damage responses.

## Discussion

Pyrazole-tethered heterocyclic compounds, such as crizotinib and ruxolitinib, have received considerable attention owing to their chemotherapeutic potential (45, 46). In this study, we synthesized PCW-1001, a novel pyrazole derivative, and investigated its radiosensitizing antitumor activity in breast cancer both *in vitro* and *in vivo*. PCW-1001 induced apoptosis of breast cancer cells by modulating DNA damage responses, thereby enhancing the radiation sensitivity of breast cancer cells. Therefore, our data provide a novel pyrazole derivative, PCW-1001, that exerts antitumor and radio-sensitizing activities in breast cancer.

Pyrazole is a useful lead compound to synthesize potent bioactive molecules for drug development with good safety profiles, particularly against different types of cancers (47). Anticancer activities of several pyrazole derivatives have been demonstrated in both *in vitro* and *in vivo* models, often resulting in promising lead compounds (47). For instance, crizotinib and ruxolitinib are important pyrazole-tethered anticancer drugs (45, 46), and celecoxib is a typical pyrazole-tethered diaryl heterocyclic small molecule with antitumor activity (15). Our data showed a novel antitumor pyrazole derivative, which introduced a sulfonylurea functional group at meta-position on the benzene ring to increase charge–charge interactions and hydrogen bonding interactions. Compound 5a, containing *the N*-ethylsulfonyl group, showed approximately 67% anticancer activity in the MCF7 cell line. The N,N-dimethylsulfonyl urea group exhibits charge–charge interactions, hydrogen bond acceptor, and hydrogen bond donor characteristics (25, 26). We substituted *the N*-ethylsulfonyl group of compound 5a with the *N,N*-dimethylsulfonyl urea group. Compound 5b showed approximately 90% inhibitory activity against the MCF7 cell line and 98% strong inhibitory activity against HCT116 cells. In addition, it exerted a strong anticancer activity against seven different cancer cell lines (**Table 1**). To confirm the importance of the *N,N*-dimethylsulfonyl urea group in compound 5a, compounds 6c and 6d were synthesized in which hydrogen atoms were substituted with a methyl group and a 4-chlorobenzyl group to eliminate intramolecular hydrogen bonding. Compared to compound 5a, compounds 6c and 6d showed decreased anticancer activity. The bulky group, 6d, with a 4-chlorobenzyl group, was reduced to a greater extent than compound 6c substituted with a small methyl group. Compounds 8a─c introduced an *N*-oxide group, with no intra-hydrogen bonding characteristics compared with the sulfonylurea group, that significantly reduced anticancer activities. Compounds 9a and 9b, containing an *N*-oxime functional group, exhibited low anticancer activity. Thus, we developed a novel antitumor pyrazole derivative containing a key functional group with *N,N*-dimethylsulfonyl urea, which exerts strong antitumor activities against seven different cancer cell lines.

Our data showed the novel pyrazole derivative, PCW-1001, had the most potent antitumor activity in multiple cancer cells, including breast, lung, colon, and brain cancers. In addition, PCW-1001 inhibited several oncogenic properties, such as cell proliferation, colony formation, and mammosphere formation, in breast cancer cells, suggesting that it could inhibit the common oncogenic pathway. We found that PCW-1001 increased apoptosis in several breast cancer cells, and programmed cell death is an essential mechanism to eliminate cancer cells by anticancer drugs (48–50). Thus, these results suggest that PCW-1001 inhibits a general oncogenic survival pathway rather than specific mutations of oncogenes or tumor suppressor genes, which are usually involved in tumor-type dependent function. In addition, PCW-1001 reduced tumor growth in breast cancer *in vivo* without remarkable changes in body weight and organ mass, including the liver, spleen, and kidneys (data not shown), suggesting low toxicity to normal cells. Therefore, these observations suggest that PCW-1001 could be a potential anticancer compound for human breast cancer.

It is well established that anticancer treatments, such as chemotherapy and radiotherapy, induce DNA damage directly or indirectly in active proliferating cancer cells rather than non-proliferating normal cells (51, 52). Our data showed that PCW-1001 modulated DNA damage response/repair genes in breast cancer cells, suggesting that PCW-1001 may induce DNA damage response in breast cancer cells. However, our data indicated that PCW-1001 alone did not significantly induce DNA damage, as evidenced by the slight increase in γ-H2AX and phospho-Chk2 levels, which are markers for DNA damage (53), compared to gamma irradiation (**Figures 6B, C**). The expression of MCM5 and MCM7, which are important regulator of DNA replication (54), is increased after PCW-1001 treatment. We found that PCW-1001 arrests cells in the G1 phase, and that MCM5 and MCM7 are upregulated in G1 (54). PCW-1001 may affect DNA damage as well as DNA replication, which is why the expression of the MCM family is changed by PCW-1001. Therefore, it remains unclear whether PCW-1001 induces DNA damage or replication stress. Although the exact mechanism of PCW-1001 is not verified, we believe that PCW-1001 induces cellular stress, such as endoplasmic reticulum (ER) stress and energy stress, instead of direct DNA damage. For example, our transcriptome analysis showed that PCW-1001 increased the expression of several stress-induced proteins such as DDIT-3, also known as CHOP (55), by over 10 folds. CHOP, a key pro-apoptotic transcription factor, is activated by ER stress or the unfolded protein response and induces apoptosis of cells *via* several apoptotic pathways (56). Due to the dramatic induction of CHOP by PCW-1001, we evaluated whether the anticancer activity of PCW-1001 is dependent on CHOP in MCF7 cells. However, siRNA-mediated depletion of *CHOP* did not prevent the anticancer activity of PCW-1001 in MCF7 cells (data not shown), indicating that the anticancer activity of PCW-1001 was independent of CHOP. In addition, the radiation sensitivity of tumor tissue in response to radiotherapy is usually regulated by DNA repair, cell cycle, re-oxygenation, and repopulation (57). Regarding the modulation of DNA damage response by PCW-1001, our data showed that PCW-1001 enhanced the radiation sensitivity of breast cancer cells by inducing DNA damage in breast cancer cells. Additional details regarding the mechanism of PCW-1001 should be verified, and we aim to identify its specific target and mechanism in further studies. Therefore, PCW-1001 could be useful not only as an anticancer molecule, but also as a potential radiosensitizer by modulating the DNA repair process during radiotherapy.

In conclusion, we synthesized and studied a novel anticancer pyrazole derivative, PCW-1001, for radiosensitizer activity using both *in vitro* and *in vivo* breast cancer models.

## Data Availability Statement

The datasets presented in this study can be found in online repositories. The names of the repository/repositories and accession number(s) can be found below: https://www.ncbi.nlm.nih.gov/geo/, GSE190883.

## Ethics Statement

The animal study was reviewed and approved by The Korea Institute of Radiological and Medical Sciences (KIRAMS) Institutional Animal Care and Use Committee (IACUC).

## Author Contributions

A-YK, MK, JO, K-YJ, and J-SK: conceived/designed experiments. A-YK, MK, and Y-JK: performed the experiments. NP, SC, KL, and K-YJ: synthesized the chemicals. A-YK, K-YJ, JO, and J-SK: analyzed the data. JA, SW, and JO: provided advice. A-YK, K-YJ, and J-SK: wrote the manuscript. All authors contributed to the article and approved the submitted version.

## Funding

This study was supported by a grant from the Korea Institute of Radiological and Medical Sciences (KIRAMS), Korea Research Institute of Chemical Technology (KRICT) funded by the Ministry of Science and ICT (MSIT), Republic of Korea (No. 50531-2021, No. SI2131-10), and the National Research Foundation of Korea (NRF-2020M2D9A2094144, NRF-2020M2D9A2094158).

## Conflict of Interest

Author SW was employed by Pharmcadd.

The remaining authors declare that the research was conducted in the absence of any commercial or financial relationships that could be construed as a potential conflict of interest.

## Publisher’s Note

All claims expressed in this article are solely those of the authors and do not necessarily represent those of their affiliated organizations, or those of the publisher, the editors and the reviewers. Any product that may be evaluated in this article, or claim that may be made by its manufacturer, is not guaranteed or endorsed by the publisher.

## Supplementary Material

Supplementary Figure 1
**(A)** Quantification of immunoblotting (**Figure 3E**). **(B)** Cell cycle analysis with PCW-1001 treatment. **(C)** Quantification of immunoblotting (**Figures 4C–E**).

Supplementary Figure 2
**(A)** The combination index of PCW-1001 and irradiation in breast cancer cells. **(B)** Immunofluorescent staining of γH2AX expression (**Figure 6B**). Scale bars = 20 µm. **(C)** Quantification of immunoblotting (**Figure 6C**).
