# Supplementary figures and images for: PCW-1001, a Novel Pyrazole Derivative, Exerts Antitumor and Radio-Sensitizing Activities in Breast Cancer
Source: Front Oncol. 2022 Mar 29;12:835833. doi: 10.3389/fonc.2022.835833 (PMC9002139; doi:10.3389/fonc.2022.835833)

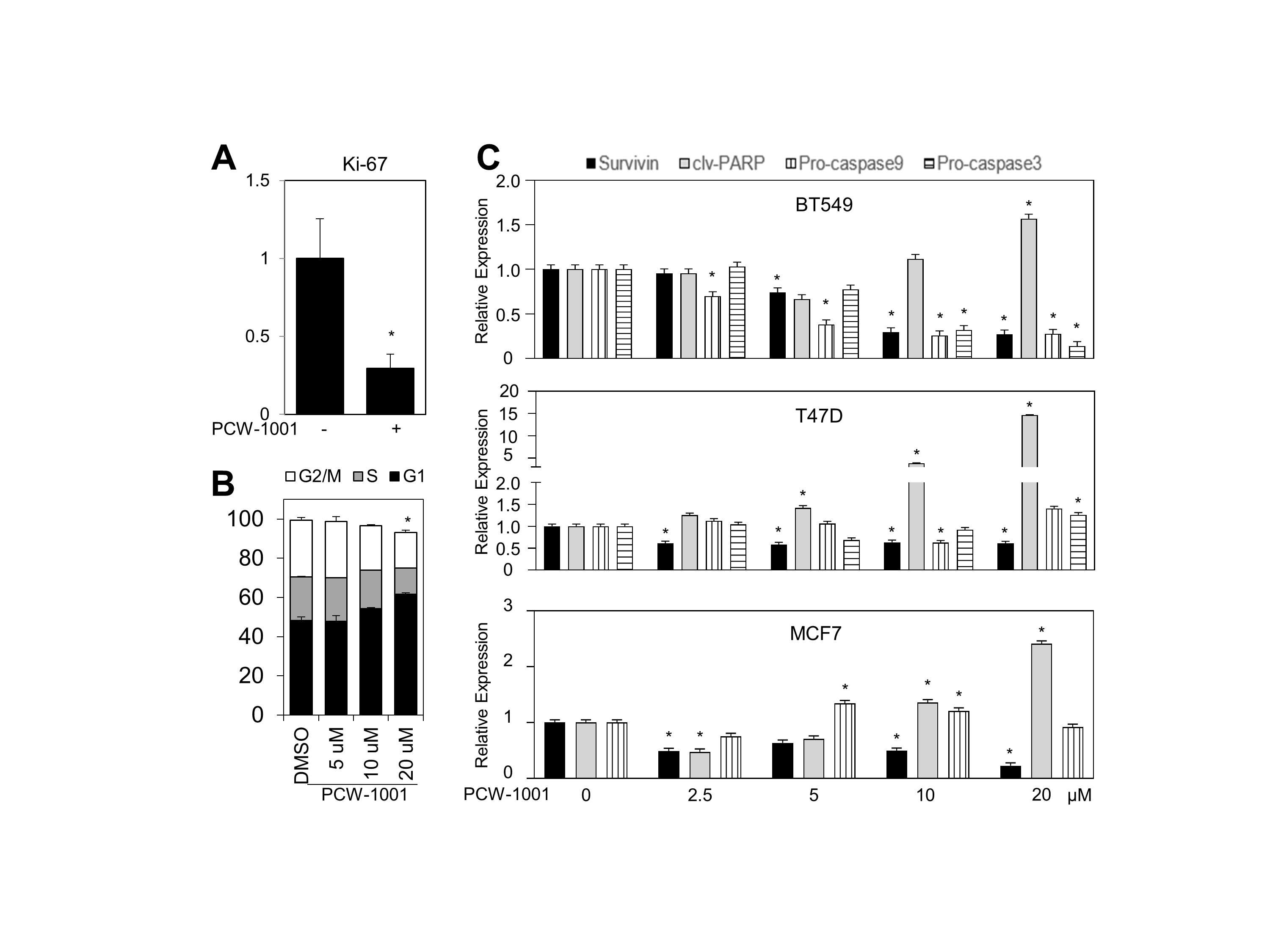

Supplement: Supplementary Figure 1 — (A) Quantification of immunoblotting ( Figure 3E ). (B) Cell cycle analysis with PCW-1001 treatment. (C) Quantification of immunoblotting ( Figures 4C–E ). [file Image_1.tif]

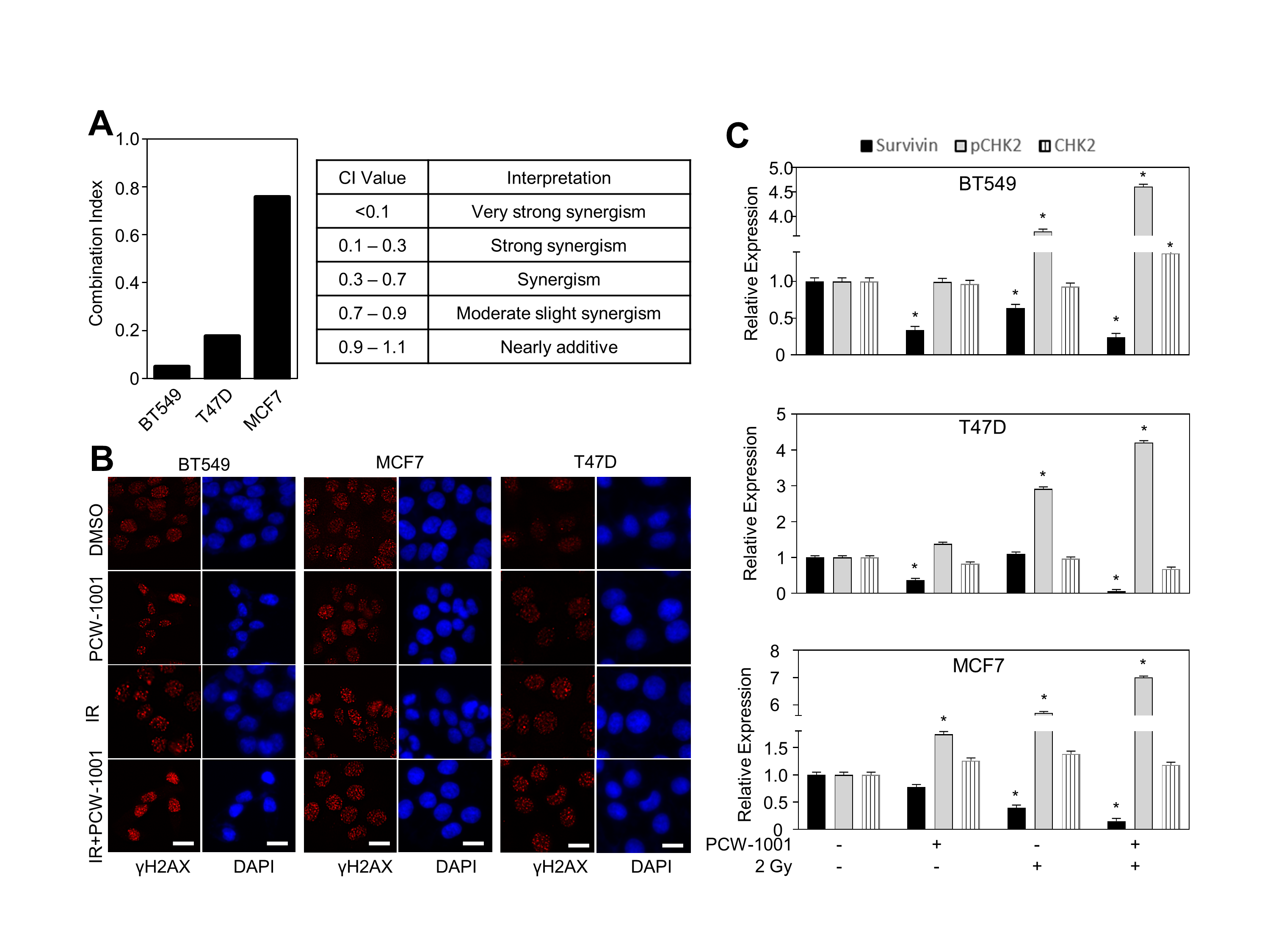

Supplement: Supplementary Figure 2 — (A) The combination index of PCW-1001 and irradiation in breast cancer cells. (B) Immunofluorescent staining of γH2AX expression ( Figure 6B ). Scale bars = 20 µm. (C) Quantification of immunoblotting ( Figure 6C ). [file Image_2.tif]
