# Supplementary material for: PCW-1001, a Novel Pyrazole Derivative, Exerts Antitumor and Radio-Sensitizing Activities in Breast Cancer
Source: Front Oncol. 2022 Mar 29;12:835833. doi: 10.3389/fonc.2022.835833 (PMC9002139; doi:10.3389/fonc.2022.835833)
Supplement: Supplementary file 3 [file Table_1.docx]

|  | BT549 | T47D | MCF7 |
| --- | --- | --- | --- |
| CTRL | 1 | 1 | 1 |
| PCW-1001 2.5 µM | 0.75 | 0.41 | 0.77 |
| IR 2Gy | 0.35 | 0.50 | 0.42 |
| PCW-1001 2.5 µM + IR 2Gy | 0.14 | 0.17 | 0.22 |

Suppl. Table 1. Survival fraction
