# Supplementary material for: PCW-1001, a Novel Pyrazole Derivative, Exerts Antitumor and Radio-Sensitizing Activities in Breast Cancer
Source: Front Oncol. 2022 Mar 29;12:835833. doi: 10.3389/fonc.2022.835833 (PMC9002139; doi:10.3389/fonc.2022.835833)

Figure 3. E

Ki-67: 359kDa

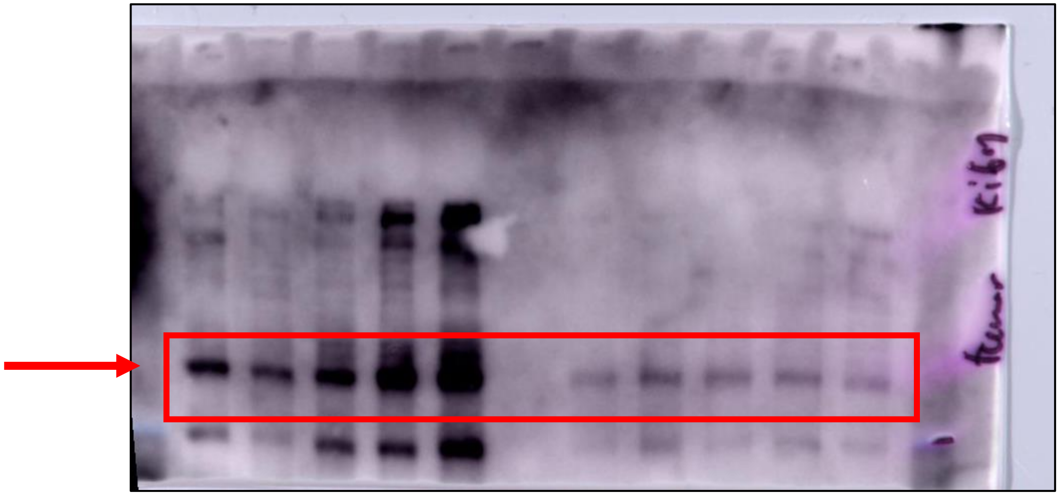

β-actin: 43kDa

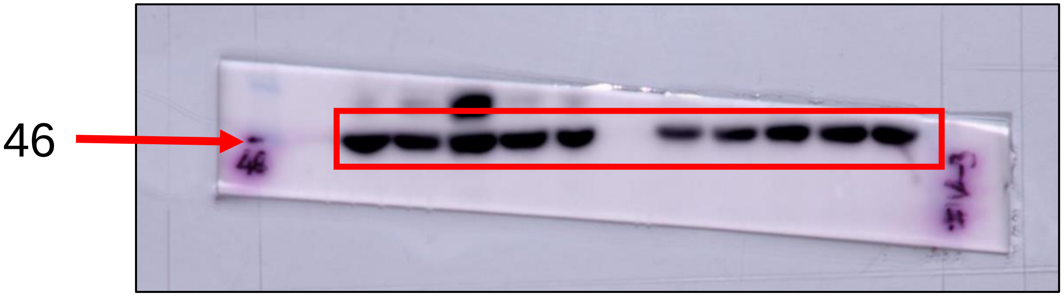

Figure 4. C

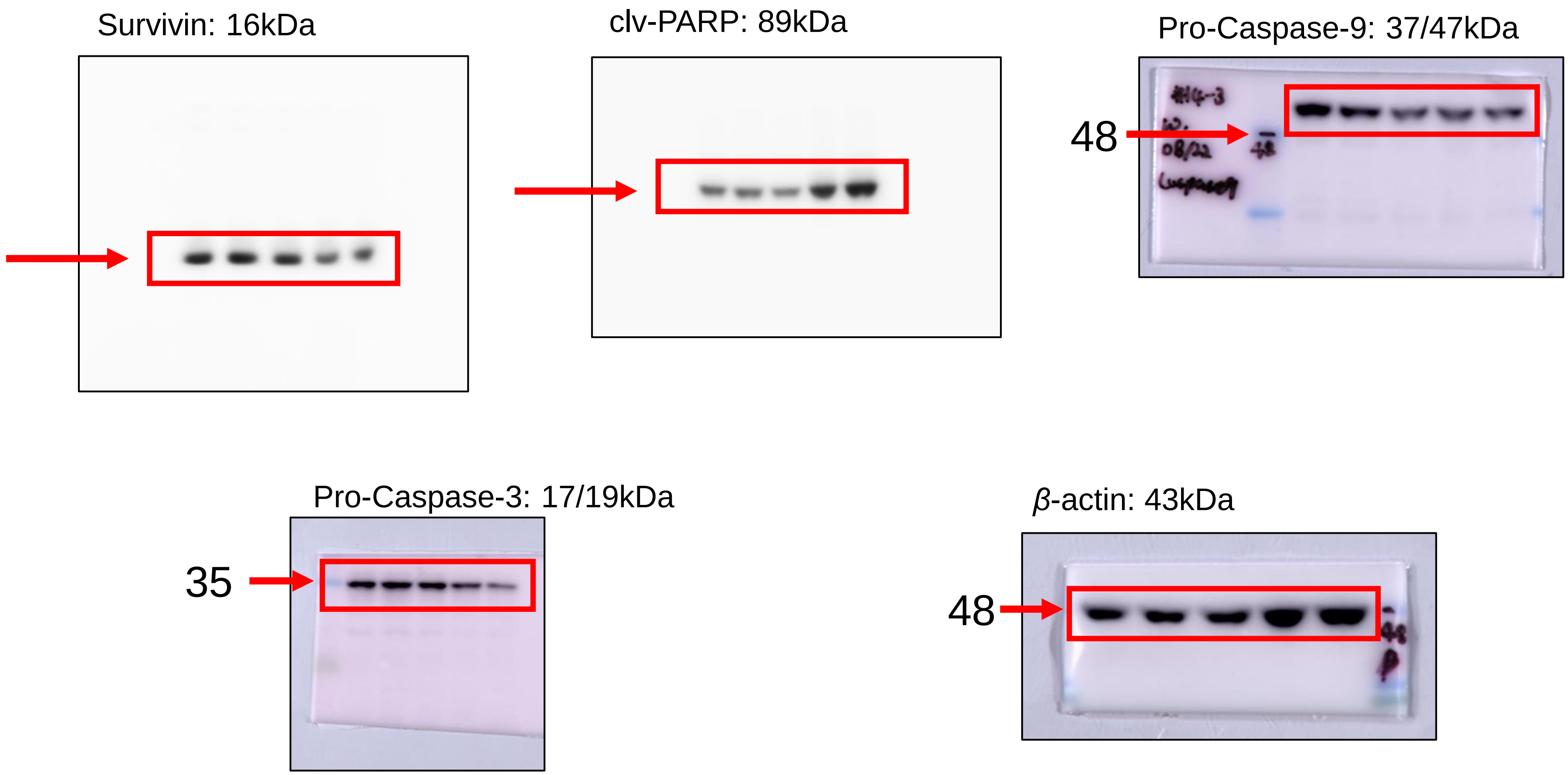
Survivin: 16kDa
clv-PARP: 89kDa
Pro-Caspase-9: 37/47kDa
48
Pro-Caspase-3: 17/19kDa
35
β-actin: 43kDa
48

Figure 4. D

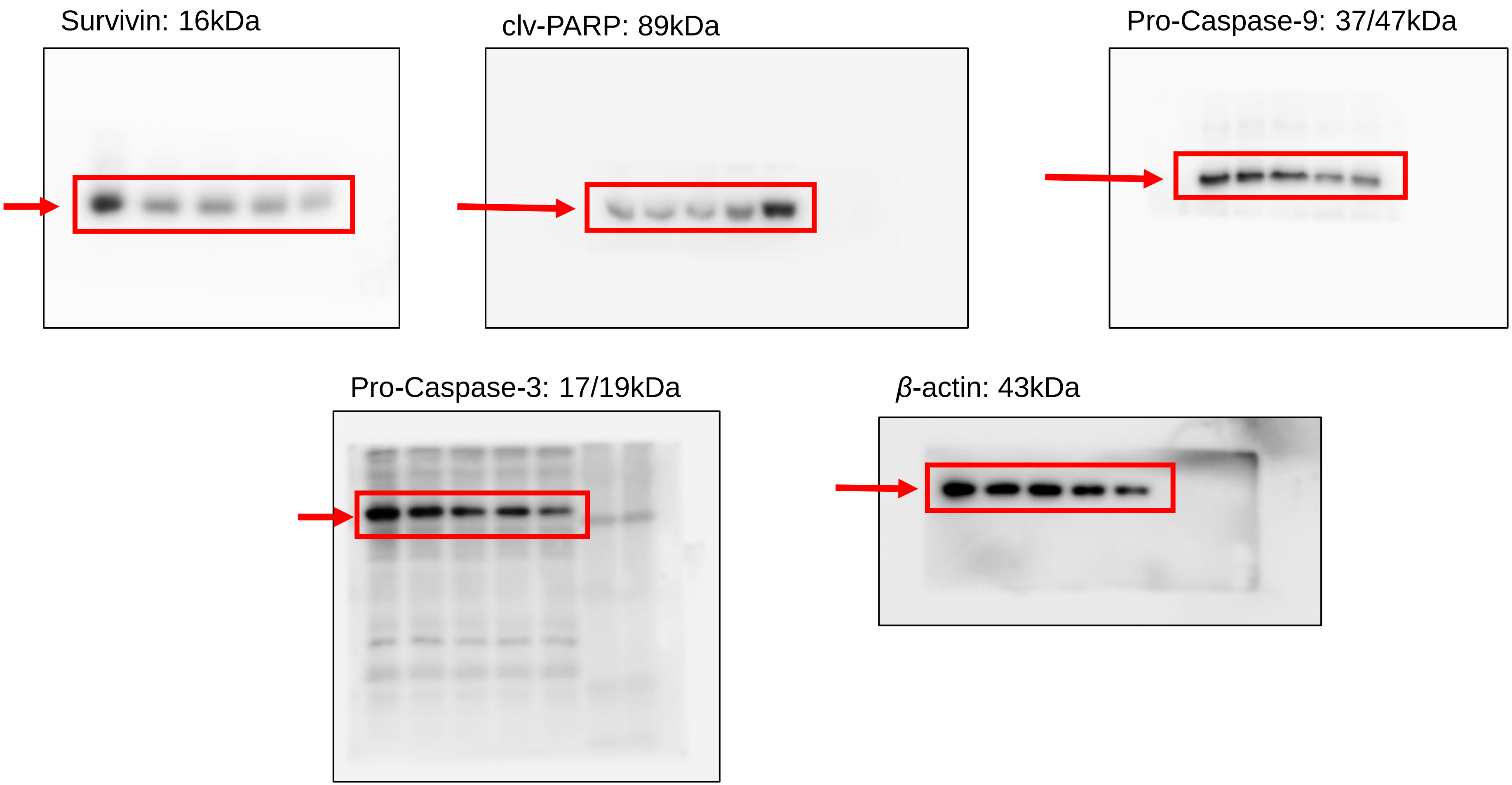

Survivin: 16kDa
clv-PARP: 89kDa
Pro-Caspase-9: 37/47kDa
Pro-Caspase-3: 17/19kDa
β-actin: 43kDa

Figure 4. E

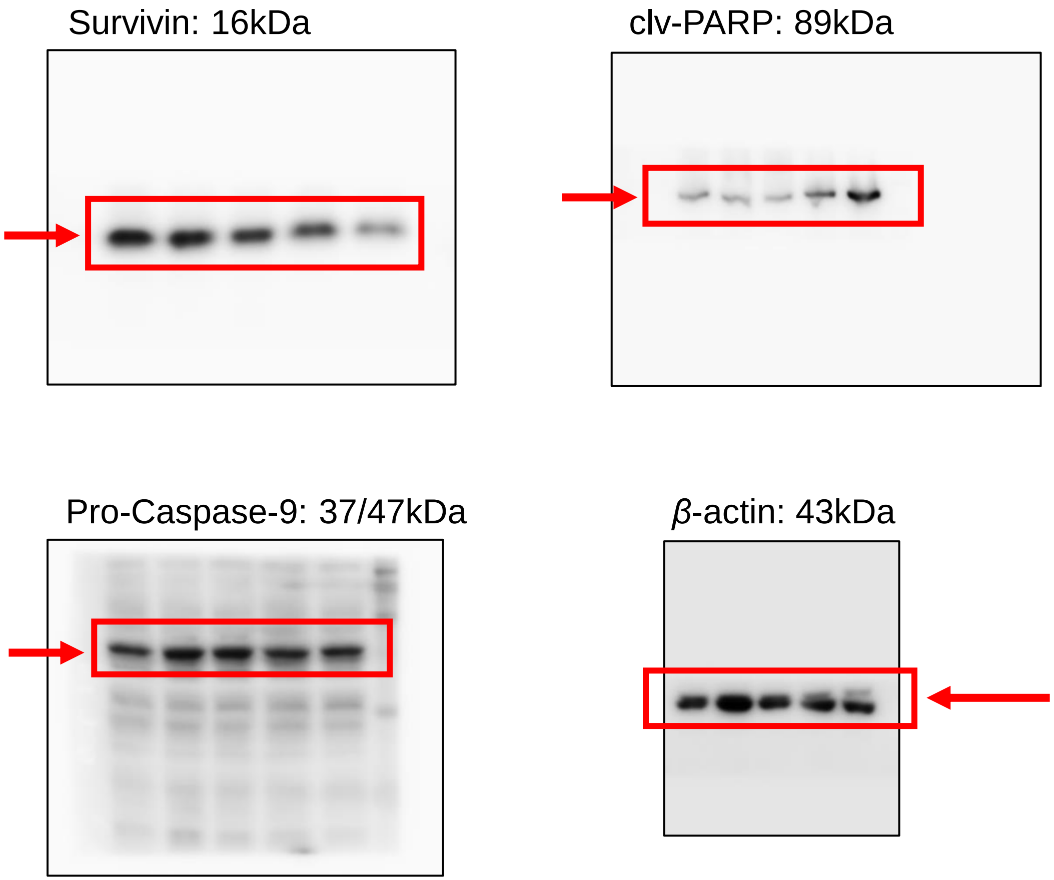
Survivin: 16kDa
clv-PARP: 89kDa
Pro-Caspase-9: 37/47kDa
β-actin: 43kDa

Figure 6. C (BT-549)

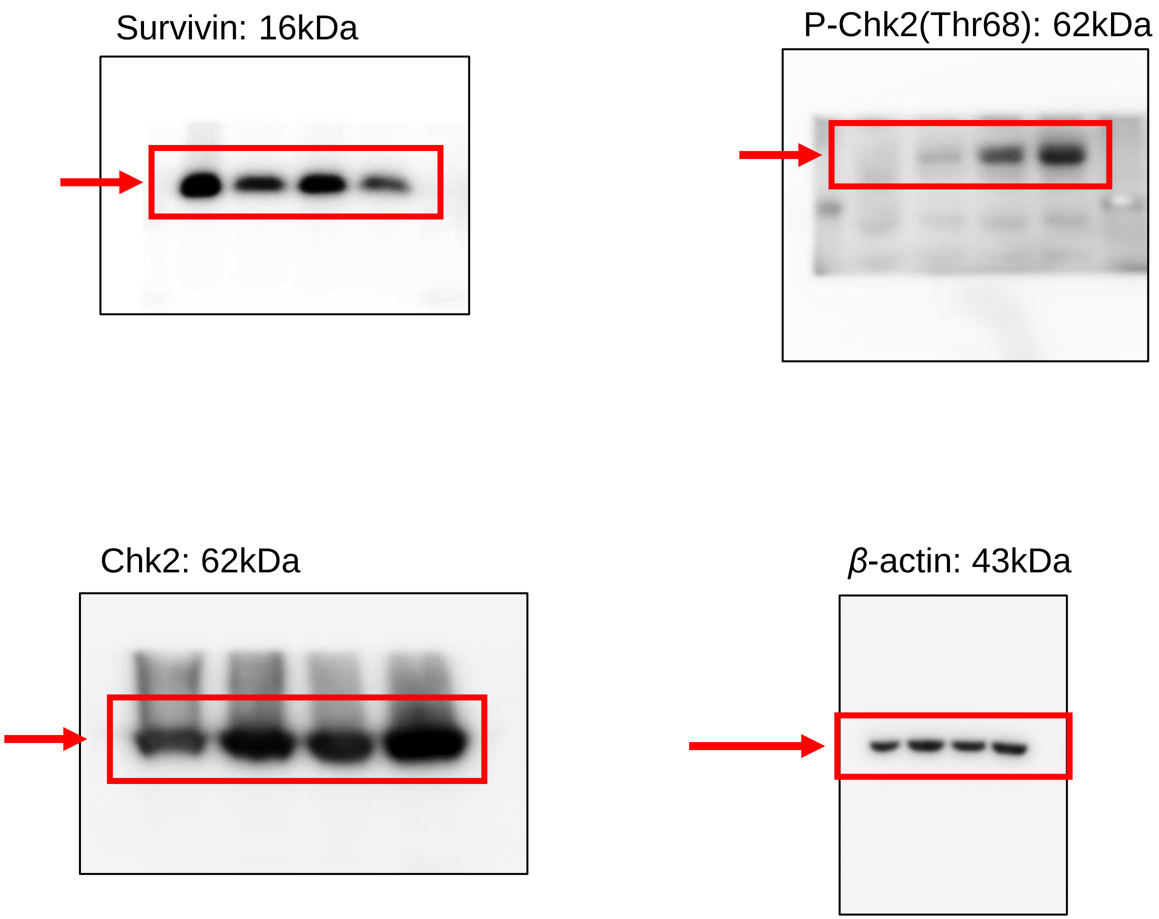

Figure 6. C (T47D)

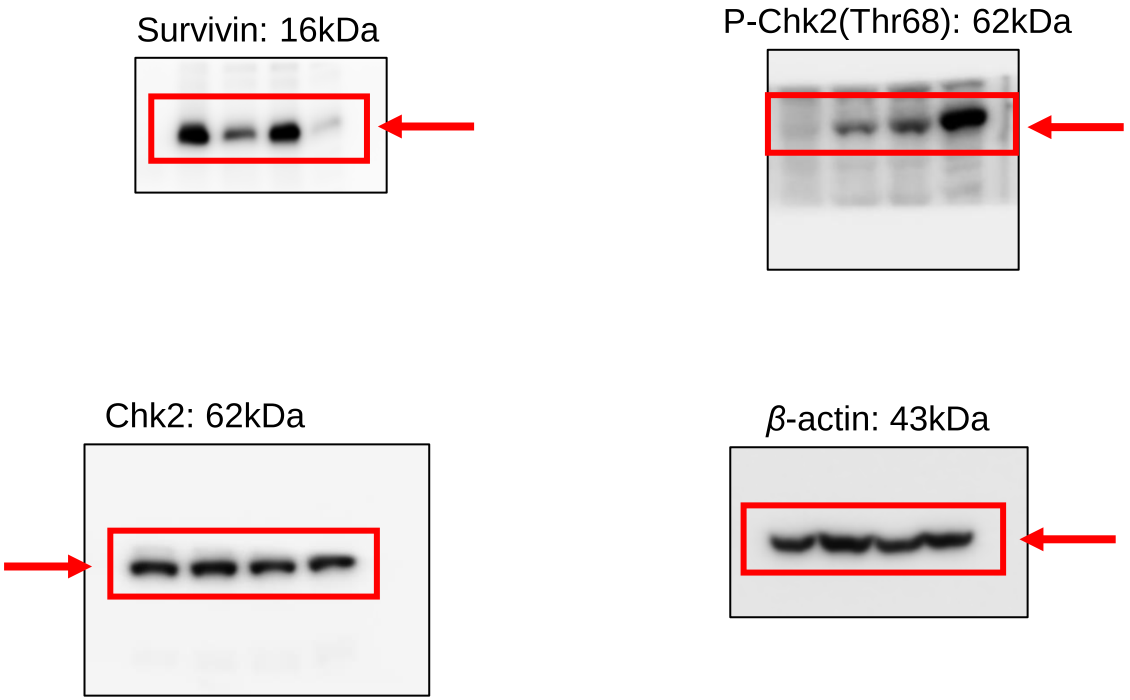

Figure 6. C (MCF7)

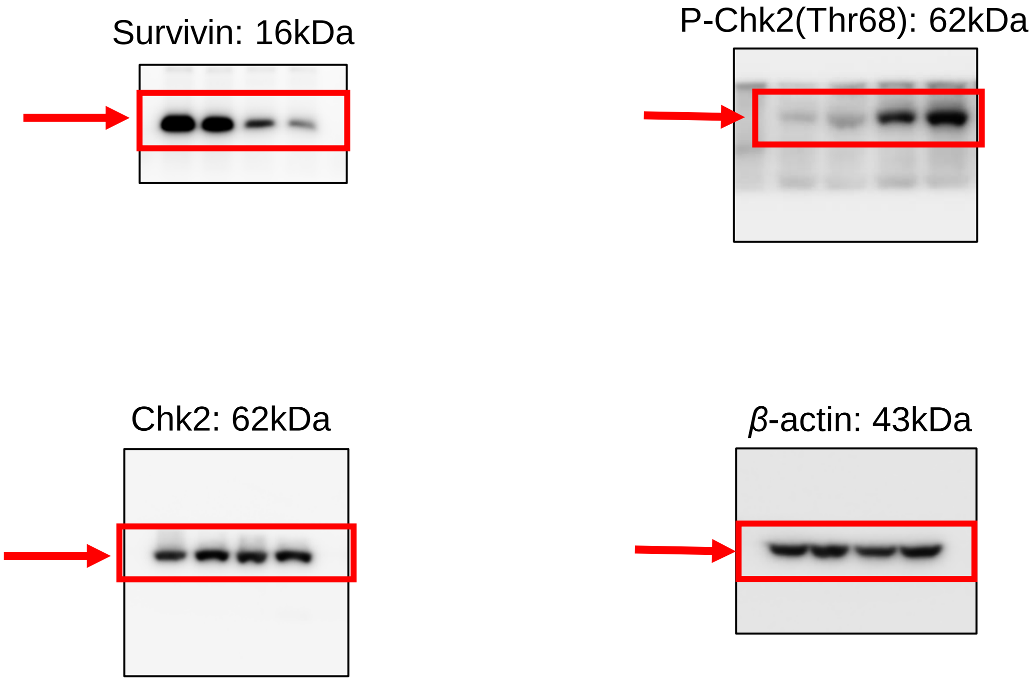

Supplement: Supplementary file 4 [file DataSheet_1.pdf]
